# Phosphorus in Neuralgia

**Published:** 1873-10

**Authors:** 


### PHOSPHORUS IN NEURALGIA.

In the *Practitioner*, for July, 1873, Dr. J. A. Thompson has an article on phosphorus in neuralgia. He records eighteen cases, and arranges them in three classes: "acute primary attacks, acute recurrent attacks and chronic cases." Six cases occur in each class. In the first class the ages ranged between twenty-five and forty-six; in the second between thirty and sixty; in the third between twenty-four and forty. Some of the patients suffered with trigeminal, some from cervico occipital, some from cervico-brachial neuralgia; and one in the second class from sciatica. All the cases in the first two classes were cured. Of the third class, three were cured (one patient had been afflicted sixteen years without a week's freedom from pain); two (both consumptive) were relieved; and one uncomplicated case, a woman, aged forty, with affection of the fifth nerve, of ten month's duration, was unbenefited, although she was treated for fifteen days. As might be expected, the chronic cases take the longest to cure, but, in all the cases benefited, relief followed the first few doses.

The author employs large doses. He says, "To prescribe less than one-twentieth of a grain in the first place is to render its therapeutic action apparently variable or uncertain." He now invariably gives one-twelfth of a grain every four hours.

He has employed phosphorus in various combinations, dissolved in oil. in ether, in chloroform, in spirit, and prefers a tincture made by dissolving the phosphorus in absolute alcohol with the assistance of heat. He says, "The most convenient proportion for dispensing is, phosphorus one grain; absolute alcohol three drachms. This mixture will, I know, retain its powers for six weeks." The following is the method of employing it: Tincture of phosphorus, three drachms; rectified spirit, two drachms; spirits of peppermint, half a drachm; water to six ounces.

This mixture being unstable, should be compounded every day. The author prefers the above form to capsules or pills.

[Dr. Percy's alkaloidal form of phosphorus, described in the last volume of the *Transactions American Medical Association*, 1872, would probably answer better.—EDS. REPORTER.]

